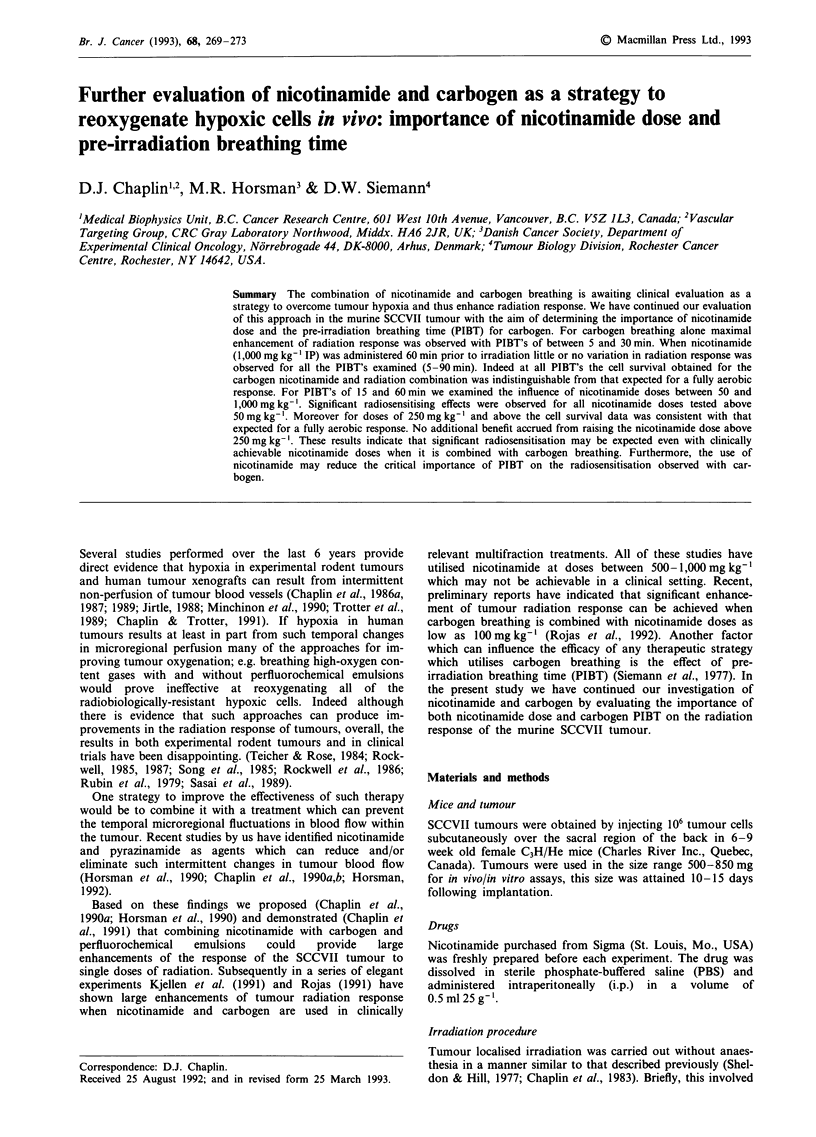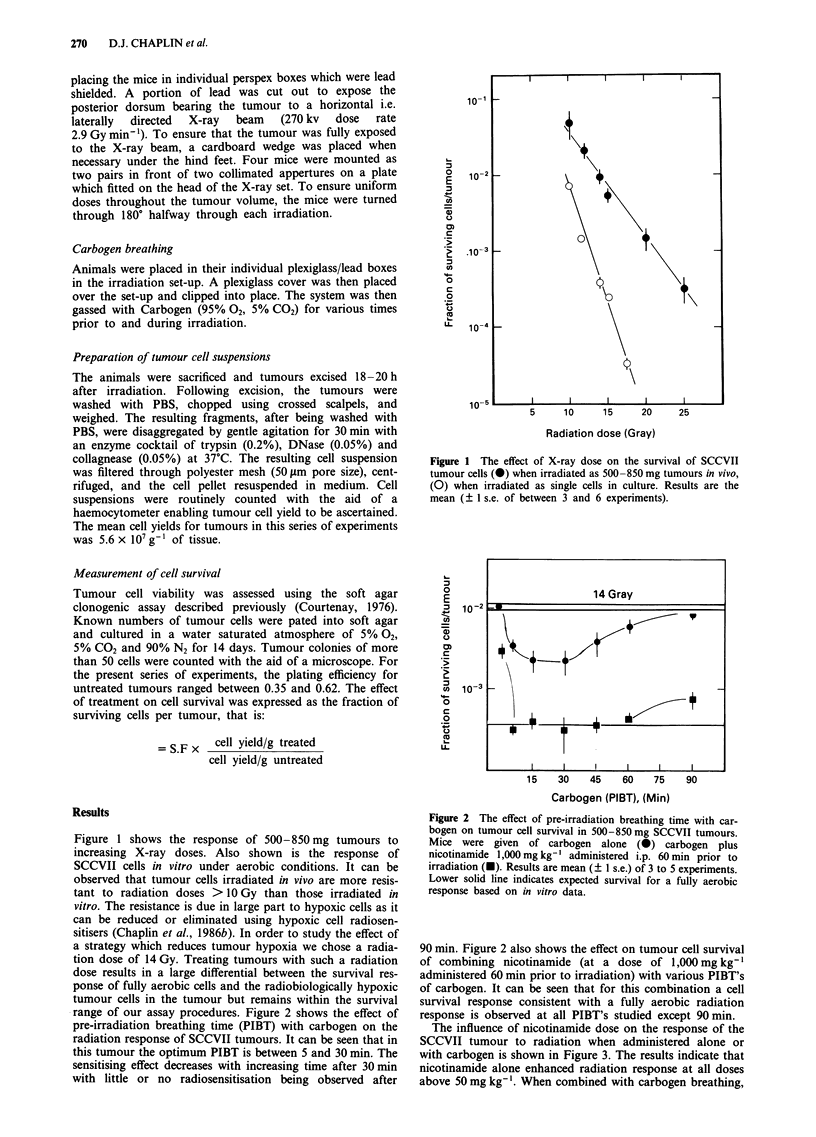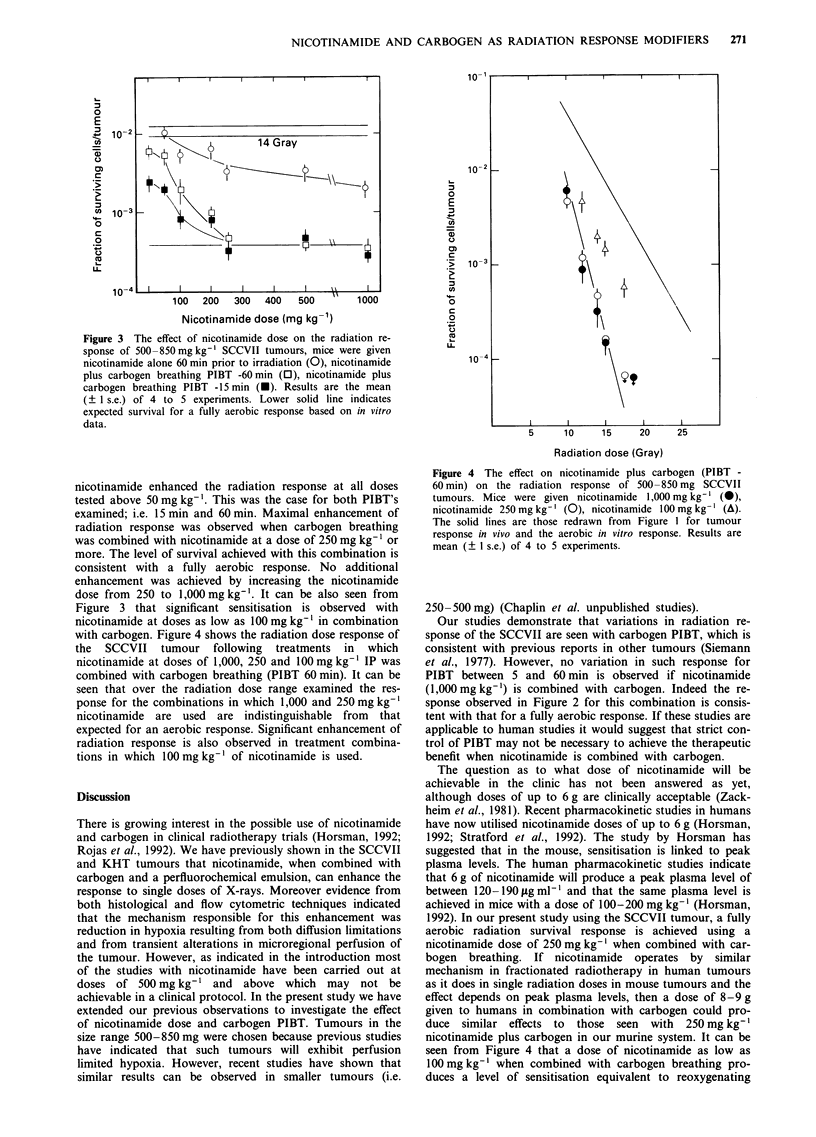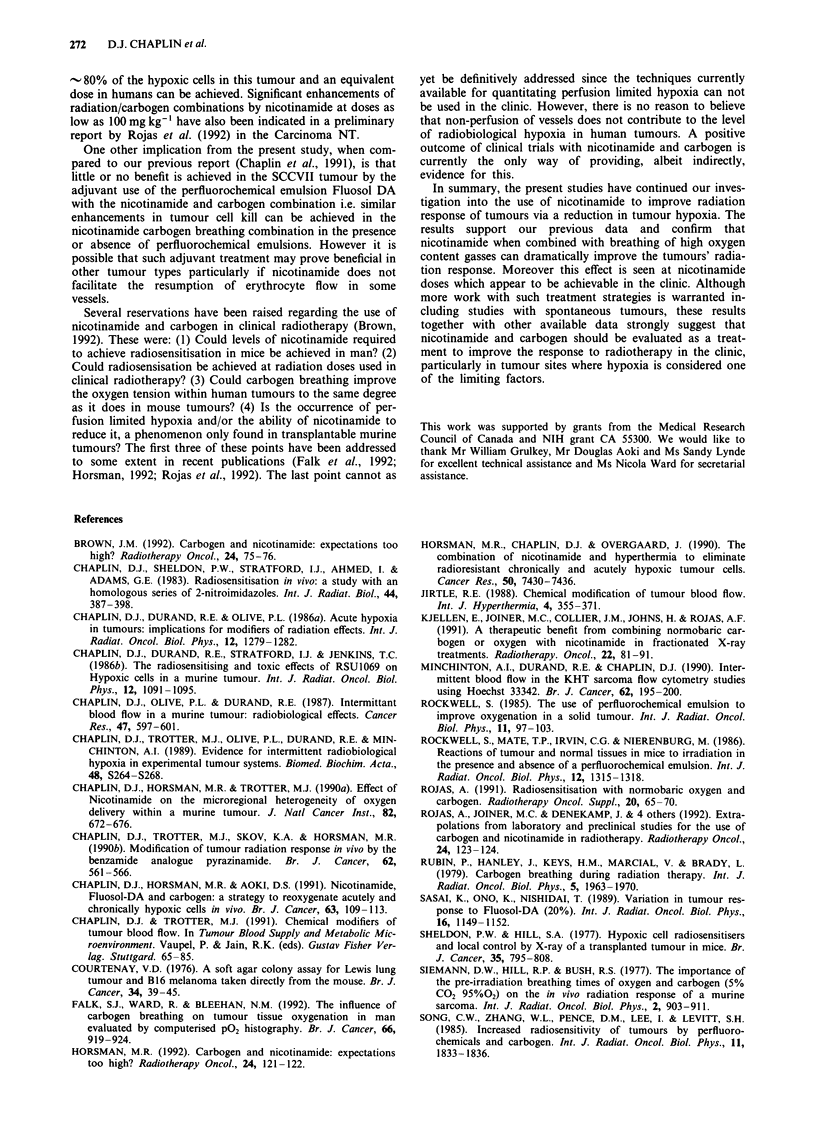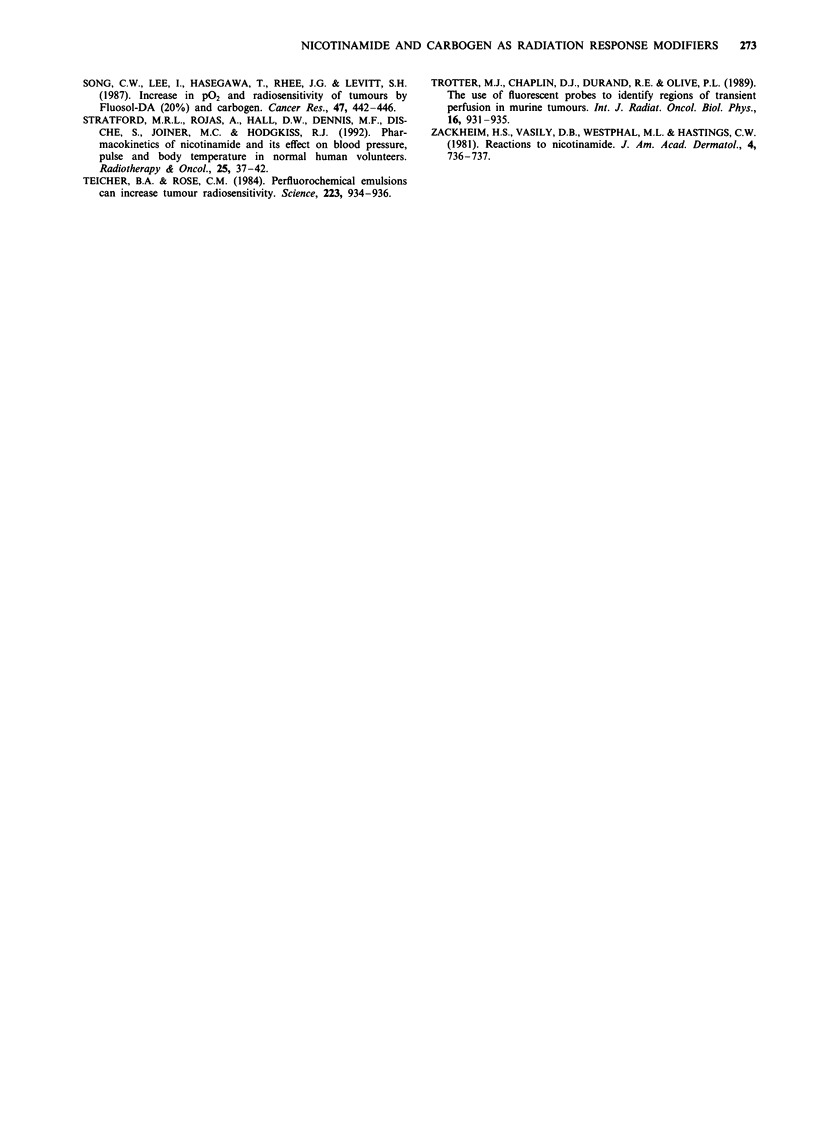# Further evaluation of nicotinamide and carbogen as a strategy to reoxygenate hypoxic cells in vivo: importance of nicotinamide dose and pre-irradiation breathing time.

**DOI:** 10.1038/bjc.1993.326

**Published:** 1993-08

**Authors:** D. J. Chaplin, M. R. Horsman, D. W. Siemann

**Affiliations:** Medical Biophysics Unit, B.C. Cancer Research Centre, Vancouver, Canada.

## Abstract

The combination of nicotinamide and carbogen breathing is awaiting clinical evaluation as a strategy to overcome tumour hypoxia and thus enhance radiation response. We have continued our evaluation of this approach in the murine SCCVII tumour with the aim of determining the importance of nicotinamide dose and the pre-irradiation breathing time (PIBT) for carbogen. For carbogen breathing alone maximal enhancement of radiation response was observed with PIBT's of between 5 and 30 min. When nicotinamide (1,000 mg kg-1 IP) was administered 60 min prior to irradiation little or no variation in radiation response was observed for all the PIBT's examined (5-90 min). Indeed at all PIBT's the cell survival obtained for the carbogen nicotinamide and radiation combination was indistinguishable from that expected for a fully aerobic response. For PIBT's of 15 and 60 min we examined the influence of nicotinamide doses between 50 and 1,000 mg kg-1. Significant radiosensitizing effects were observed for all nicotinamide doses tested above 50 mg kg-1. Moreover for doses of 250 mg kg-1 and above the cell survival data was consistent with that expected for a fully aerobic response. No additional benefit accrued from raising the nicotinamide dose above 250 mg kg-1. These results indicate that significant radiosensitization may be expected even with clinically achievable nicotinamide doses when it is combined with carbogen breathing. Furthermore, the use of nicotinamide may reduce the critical importance of PIBT on the radiosensitization observed with carbogen.


Br. J. Cancer (1993), 68, 269-273 © Macmillan Press Ltd., 1993

### Further evaluation of nicotinamide and carbogen as a strategy to reoxygenate hypoxic cells *in vivo*: importance of nicotinamide dose and pre-irradiation breathing time

D.J. Chaplin[1,2], M.R. Horsman[3] & D.W. Siemann[4]

[1]*Medical Biophysics Unit, B.C. Cancer Research Centre, 601 West 10th Avenue, Vancouver, B.C. V5Z 1L3, Canada;* [2]*Vascular Targeting Group, CRC Gray Laboratory Northwood, Middx. HA6 2JR, UK;* [3]*Danish Cancer Society, Department of Experimental Clinical Oncology, Nörrebrogade 44, DK-8000, Arhus, Denmark;* [4]*Tumour Biology Division, Rochester Cancer Centre, Rochester, NY 14642, USA.*

**Summary** The combination of nicotinamide and carbogen breathing is awaiting clinical evaluation as a strategy to overcome tumour hypoxia and thus enhance radiation response. We have continued our evaluation of this approach in the murine SCCVII tumour with the aim of determining the importance of nicotinamide dose and the pre-irradiation breathing time (PIBT) for carbogen. For carbogen breathing alone maximal enhancement of radiation response was observed with PIBT's of between 5 and 30 min. When nicotinamide (1,000 mg $kg^{-1}$ IP) was administered 60 min prior to irradiation little or no variation in radiation response was observed for all the PIBT's examined (5-90 min). Indeed at all PIBT's the cell survival obtained for the carbogen nicotinamide and radiation combination was indistinguishable from that expected for a fully aerobic response. For PIBT's of 15 and 60 min we examined the influence of nicotinamide doses between 50 and 1,000 mg $kg^{-1}$. Significant radiosensitising effects were observed for all nicotinamide doses tested above 50 mg $kg^{-1}$. Moreover for doses of 250 mg $kg^{-1}$ and above the cell survival data was consistent with that expected for a fully aerobic response. No additional benefit accrued from raising the nicotinamide dose above 250 mg $kg^{-1}$. These results indicate that significant radiosensitisation may be expected even with clinically achievable nicotinamide doses when it is combined with carbogen breathing. Furthermore, the use of nicotinamide may reduce the critical importance of PIBT on the radiosensitisation observed with carbogen.

Several studies performed over the last 6 years provide direct evidence that hypoxia in experimental rodent tumours and human tumour xenografts can result from intermittent non-perfusion of tumour blood vessels (Chaplin *et al.*, 1986*a*, 1987; 1989; Jirtle, 1988; Minchinon *et al.*, 1990; Trotter *et al.*, 1989; Chaplin & Trotter, 1991). If hypoxia in human tumours results at least in part from such temporal changes in microregional perfusion many of the approaches for improving tumour oxygenation; e.g. breathing high-oxygen content gases with and without perfluorochemical emulsions would prove ineffective at reoxygenating all of the radiobiologically-resistant hypoxic cells. Indeed although there is evidence that such approaches can produce improvements in the radiation response of tumours, overall, the results in both experimental rodent tumours and in clinical trials have been disappointing. (Teicher & Rose, 1984; Rockwell, 1985, 1987; Song *et al.*, 1985; Rockwell *et al.*, 1986; Rubin *et al.*, 1979; Sasai *et al.*, 1989).

One strategy to improve the effectiveness of such therapy would be to combine it with a treatment which can prevent the temporal microregional fluctuations in blood flow within the tumour. Recent studies by us have identified nicotinamide and pyrazinamide as agents which can reduce and/or eliminate such intermittent changes in tumour blood flow (Horsman *et al.*, 1990; Chaplin *et al.*, 1990*a*,*b*; Horsman, 1992).

Based on these findings we proposed (Chaplin *et al.*, 1990*a*; Horsman *et al.*, 1990) and demonstrated (Chaplin *et al.*, 1991) that combining nicotinamide with carbogen and perfluorochemical emulsions could provide large enhancements of the response of the SCCVII tumour to single doses of radiation. Subsequently in a series of elegant experiments Kjellen *et al.* (1991) and Rojas (1991) have shown large enhancements of tumour radiation response when nicotinamide and carbogen are used in clinically relevant multifraction treatments. All of these studies have utilised nicotinamide at doses between 500-1,000 mg $kg^{-1}$ which may not be achievable in a clinical setting. Recent, preliminary reports have indicated that significant enhancement of tumour radiation response can be achieved when carbogen breathing is combined with nicotinamide doses as low as 100 mg $kg^{-1}$ (Rojas *et al.*, 1992). Another factor which can influence the efficacy of any therapeutic strategy which utilises carbogen breathing is the effect of pre-irradiation breathing time (PIBT) (Siemann *et al.*, 1977). In the present study we have continued our investigation of nicotinamide and carbogen by evaluating the importance of both nicotinamide dose and carbogen PIBT on the radiation response of the murine SCCVII tumour.

### Materials and methods

### *Mice and tumour*

SCCVII tumours were obtained by injecting $10^6$ tumour cells subcutaneously over the sacral region of the back in 6-9 week old female $C_3H$/He mice (Charles River Inc., Quebec, Canada). Tumours were used in the size range 500-850 mg for *in vivo*/*in vitro* assays, this size was attained 10-15 days following implantation.

### *Drugs*

Nicotinamide purchased from Sigma (St. Louis, Mo., USA) was freshly prepared before each experiment. The drug was dissolved in sterile phosphate-buffered saline (PBS) and administered intraperitoneally (i.p.) in a volume of 0.5 ml 25 $g^{-1}$.

### *Irradiation procedure*

Tumour localised irradiation was carried out without anaesthesia in a manner similar to that described previously (Sheldon & Hill, 1977; Chaplin *et al.*, 1983). Briefly, this involved

Correspondence: D.J. Chaplin.
Received 25 August 1992; and in revised form 25 March 1993.

placing the mice in individual perspex boxes which were lead shielded. A portion of lead was cut out to expose the posterior dorsum bearing the tumour to a horizontal i.e. laterally directed X-ray beam (270 kv dose rate 2.9 Gy $min^{-1}$). To ensure that the tumour was fully exposed to the X-ray beam, a cardboard wedge was placed when necessary under the hind feet. Four mice were mounted as two pairs in front of two collimated appertures on a plate which fitted on the head of the X-ray set. To ensure uniform doses throughout the tumour volume, the mice were turned through 180° halfway through each irradiation.

*Carbogen breathing*

Animals were placed in their individual plexiglass/lead boxes in the irradiation set-up. A plexiglass cover was then placed over the set-up and clipped into place. The system was then gassed with Carbogen (95% $O_2$, 5% $CO_2$) for various times prior to and during irradiation.

*Preparation of tumour cell suspensions*

The animals were sacrificed and tumours excised 18–20 h after irradiation. Following excision, the tumours were washed with PBS, chopped using crossed scalpels, and weighed. The resulting fragments, after being washed with PBS, were disaggregated by gentle agitation for 30 min with an enzyme cocktail of trypsin (0.2%), DNase (0.05%) and collagnease (0.05%) at 37°C. The resulting cell suspension was filtered through polyester mesh (50 μm pore size), centrifuged, and the cell pellet resuspended in medium. Cell suspensions were routinely counted with the aid of a haemocytometer enabling tumour cell yield to be ascertained. The mean cell yields for tumours in this series of experiments was $5.6 \times 10^7\ g^{-1}$ of tissue.

*Measurement of cell survival*

Tumour cell viability was assessed using the soft agar clonogenic assay described previously (Courtenay, 1976). Known numbers of tumour cells were pated into soft agar and cultured in a water saturated atmosphere of 5% $O_2$, 5% $CO_2$ and 90% $N_2$ for 14 days. Tumour colonies of more than 50 cells were counted with the aid of a microscope. For the present series of experiments, the plating efficiency for untreated tumours ranged between 0.35 and 0.62. The effect of treatment on cell survival was expressed as the fraction of surviving cells per tumour, that is:

$$= S.F \times \frac{\text{cell yield/g treated}}{\text{cell yield/g untreated}}$$

### Results

Figure 1 shows the response of 500–850 mg tumours to increasing X-ray doses. Also shown is the response of SCCVII cells *in vitro* under aerobic conditions. It can be observed that tumour cells irradiated *in vivo* are more resistant to radiation doses $>$10 Gy than those irradiated *in vitro*. The resistance is due in large part to hypoxic cells as it can be reduced or eliminated using hypoxic cell radiosensitisers (Chaplin *et al.*, 1986*b*). In order to study the effect of a strategy which reduces tumour hypoxia we chose a radiation dose of 14 Gy. Treating tumours with such a radiation dose results in a large differential between the survival response of fully aerobic cells and the radiobiologically hypoxic tumour cells in the tumour but remains within the survival range of our assay procedures. Figure 2 shows the effect of pre-irradiation breathing time (PIBT) with carbogen on the radiation response of SCCVII tumours. It can be seen that in this tumour the optimum PIBT is between 5 and 30 min. The sensitising effect decreases with increasing time after 30 min with little or no radiosensitisation being observed after

**Figure 1** The effect of X-ray dose on the survival of SCCVII tumour cells (●) when irradiated as 500–850 mg tumours *in vivo*, (O) when irradiated as single cells in culture. Results are the mean (± 1 s.e. of between 3 and 6 experiments).

**Figure 2** The effect of pre-irradiation breathing time with carbogen on tumour cell survival in 500–850 mg SCCVII tumours. Mice were given of carbogen alone (●) carbogen plus nicotinamide 1,000 mg $kg^{-1}$ administered i.p. 60 min prior to irradiation (■). Results are mean (± 1 s.e.) of 3 to 5 experiments. Lower solid line indicates expected survival for a fully aerobic response based on *in vitro* data.

90 min. Figure 2 also shows the effect on tumour cell survival of combining nicotinamide (at a dose of 1,000 mg $kg^{-1}$ administered 60 min prior to irradiation) with various PIBT's of carbogen. It can be seen that for this combination a cell survival response consistent with a fully aerobic radiation response is observed at all PIBT's studied except 90 min.

The influence of nicotinamide dose on the response of the SCCVII tumour to radiation when administered alone or with carbogen is shown in Figure 3. The results indicate that nicotinamide alone enhanced radiation response at all doses above 50 mg $kg^{-1}$. When combined with carbogen breathing,

**Figure 3** The effect of nicotinamide dose on the radiation response of 500–850 mg kg$^{-1}$ SCCVII tumours, mice were given nicotinamide alone 60 min prior to irradiation (○), nicotinamide plus carbogen breathing PIBT -60 min (□), nicotinamide plus carbogen breathing PIBT -15 min (■). Results are the mean (± 1 s.e.) of 4 to 5 experiments. Lower solid line indicates expected survival for a fully aerobic response based on *in vitro* data.

**Figure 4** The effect on nicotinamide plus carbogen (PIBT - 60 min) on the radiation response of 500–850 mg SCCVII tumours. Mice were given nicotinamide 1,000 mg kg$^{-1}$ (●), nicotinamide 250 mg kg$^{-1}$ (○), nicotinamide 100 mg kg$^{-1}$ (Δ). The solid lines are those redrawn from Figure 1 for tumour response *in vivo* and the aerobic *in vitro* response. Results are mean (± 1 s.e.) of 4 to 5 experiments.

nicotinamide enhanced the radiation response at all doses tested above 50 mg kg$^{-1}$. This was the case for both PIBT's examined; i.e. 15 min and 60 min. Maximal enhancement of radiation response was observed when carbogen breathing was combined with nicotinamide at a dose of 250 mg kg$^{-1}$ or more. The level of survival achieved with this combination is consistent with a fully aerobic response. No additional enhancement was achieved by increasing the nicotinamide dose from 250 to 1,000 mg kg$^{-1}$. It can be also seen from Figure 3 that significant sensitisation is observed with nicotinamide at doses as low as 100 mg kg$^{-1}$ in combination with carbogen. Figure 4 shows the radiation dose response of the SCCVII tumour following treatments in which nicotinamide at doses of 1,000, 250 and 100 mg kg$^{-1}$ IP was combined with carbogen breathing (PIBT 60 min). It can be seen that over the radiation dose range examined the response for the combinations in which 1,000 and 250 mg kg$^{-1}$ nicotinamide are used are indistinguishable from that expected for an aerobic response. Significant enhancement of radiation response is also observed in treatment combinations in which 100 mg kg$^{-1}$ of nicotinamide is used.

### Discussion

There is growing interest in the possible use of nicotinamide and carbogen in clinical radiotherapy trials (Horsman, 1992; Rojas *et al.*, 1992). We have previously shown in the SCCVII and KHT tumours that nicotinamide, when combined with carbogen and a perfluorochemical emulsion, can enhance the response to single doses of X-rays. Moreover evidence from both histological and flow cytometric techniques indicated that the mechanism responsible for this enhancement was reduction in hypoxia resulting from both diffusion limitations and from transient alterations in microregional perfusion of the tumour. However, as indicated in the introduction most of the studies with nicotinamide have been carried out at doses of 500 mg kg$^{-1}$ and above which may not be achievable in a clinical protocol. In the present study we have extended our previous observations to investigate the effect of nicotinamide dose and carbogen PIBT. Tumours in the size range 500–850 mg were chosen because previous studies have indicated that such tumours will exhibit perfusion limited hypoxia. However, recent studies have shown that similar results can be observed in smaller tumours (i.e. 250–500 mg) (Chaplin *et al.* unpublished studies).

Our studies demonstrate that variations in radiation response of the SCCVII are seen with carbogen PIBT, which is consistent with previous reports in other tumours (Siemann *et al.*, 1977). However, no variation in such response for PIBT between 5 and 60 min is observed if nicotinamide (1,000 mg kg$^{-1}$) is combined with carbogen. Indeed the response observed in Figure 2 for this combination is consistent with that for a fully aerobic response. If these studies are applicable to human studies it would suggest that strict control of PIBT may not be necessary to achieve the therapeutic benefit when nicotinamide is combined with carbogen.

The question as to what dose of nicotinamide will be achievable in the clinic has not been answered as yet, although doses of up to 6 g are clinically acceptable (Zackheim *et al.*, 1981). Recent pharmacokinetic studies in humans have now utilised nicotinamide doses of up to 6 g (Horsman, 1992; Stratford *et al.*, 1992). The study by Horsman has suggested that in the mouse, sensitisation is linked to peak plasma levels. The human pharmacokinetic studies indicate that 6 g of nicotinamide will produce a peak plasma level of between 120–190 μg ml$^{-1}$ and that the same plasma level is achieved in mice with a dose of 100–200 mg kg$^{-1}$ (Horsman, 1992). In our present study using the SCCVII tumour, a fully aerobic radiation survival response is achieved using a nicotinamide dose of 250 mg kg$^{-1}$ when combined with carbogen breathing. If nicotinamide operates by similar mechanism in fractionated radiotherapy in human tumours as it does in single radiation doses in mouse tumours and the effect depends on peak plasma levels, then a dose of 8–9 g given to humans in combination with carbogen could produce similar effects to those seen with 250 mg kg$^{-1}$ nicotinamide plus carbogen in our murine system. It can be seen from Figure 4 that a dose of nicotinamide as low as 100 mg kg$^{-1}$ when combined with carbogen breathing produces a level of sensitisation equivalent to reoxygenating

~80% of the hypoxic cells in this tumour and an equivalent dose in humans can be achieved. Significant enhancements of radiation/carbogen combinations by nicotinamide at doses as low as 100 mg $kg^{-1}$ have also been indicated in a preliminary report by Rojas *et al.* (1992) in the Carcinoma NT.

One other implication from the present study, when compared to our previous report (Chaplin *et al.*, 1991), is that little or no benefit is achieved in the SCCVII tumour by the adjuvant use of the perfluorochemical emulsion Fluosol DA with the nicotinamide and carbogen combination i.e. similar enhancements in tumour cell kill can be achieved in the nicotinamide carbogen breathing combination in the presence or absence of perfluorochemical emulsions. However it is possible that such adjuvant treatment may prove beneficial in other tumour types particularly if nicotinamide does not facilitate the resumption of erythrocyte flow in some vessels.

Several reservations have been raised regarding the use of nicotinamide and carbogen in clinical radiotherapy (Brown, 1992). These were: (1) Could levels of nicotinamide required to achieve radiosensitisation in mice be achieved in man? (2) Could radiosensisation be achieved at radiation doses used in clinical radiotherapy? (3) Could carbogen breathing improve the oxygen tension within human tumours to the same degree as it does in mouse tumours? (4) Is the occurrence of perfusion limited hypoxia and/or the ability of nicotinamide to reduce it, a phenomenon only found in transplantable murine tumours? The first three of these points have been addressed to some extent in recent publications (Falk *et al.*, 1992; Horsman, 1992; Rojas *et al.*, 1992). The last point cannot as yet be definitively addressed since the techniques currently available for quantitating perfusion limited hypoxia can not be used in the clinic. However, there is no reason to believe that non-perfusion of vessels does not contribute to the level of radiobiological hypoxia in human tumours. A positive outcome of clinical trials with nicotinamide and carbogen is currently the only way of providing, albeit indirectly, evidence for this.

In summary, the present studies have continued our investigation into the use of nicotinamide to improve radiation response of tumours via a reduction in tumour hypoxia. The results support our previous data and confirm that nicotinamide when combined with breathing of high oxygen content gasses can dramatically improve the tumours' radiation response. Moreover this effect is seen at nicotinamide doses which appear to be achievable in the clinic. Although more work with such treatment strategies is warranted including studies with spontaneous tumours, these results together with other available data strongly suggest that nicotinamide and carbogen should be evaluated as a treatment to improve the response to radiotherapy in the clinic, particularly in tumour sites where hypoxia is considered one of the limiting factors.

This work was supported by grants from the Medical Research Council of Canada and NIH grant CA 55300. We would like to thank Mr William Grulkey, Mr Douglas Aoki and Ms Sandy Lynde for excellent technical assistance and Ms Nicola Ward for secretarial assistance.

### References

BROWN, J.M. (1992). Carbogen and nicotinamide: expectations too high? *Radiotherapy Oncol.*, **24**, 75-76.

CHAPLIN, D.J., SHELDON, P.W., STRATFORD, I.J., AHMED, I. & ADAMS, G.E. (1983). Radiosensitisation *in vivo*: a study with an homologous series of 2-nitroimidazoles. *Int. J. Radiat. Biol.*, **44**, 387-398.

CHAPLIN, D.J., DURAND, R.E. & OLIVE, P.L. (1986*a*). Acute hypoxia in tumours: implications for modifiers of radiation effects. *Int. J. Radiat. Oncol. Biol. Phys.*, **12**, 1279-1282.

CHAPLIN, D.J., DURAND, R.E., STRATFORD, I.J. & JENKINS, T.C. (1986*b*). The radiosensitising and toxic effects of RSU1069 on Hypoxic cells in a murine tumour. *Int. J. Radiat. Oncol. Biol. Phys.*, **12**, 1091-1095.

CHAPLIN, D.J., OLIVE, P.L. & DURAND, R.E. (1987). Intermittant blood flow in a murine tumour: radiobiological effects. *Cancer Res.*, **47**, 597-601.

CHAPLIN, D.J., TROTTER, M.J., OLIVE, P.L., DURAND, R.E. & MINCHINTON, A.I. (1989). Evidence for intermittent radiobiological hypoxia in experimental tumour systems. *Biomed. Biochim. Acta.*, **48**, S264-S268.

CHAPLIN, D.J., HORSMAN, M.R. & TROTTER, M.J. (1990*a*). Effect of Nicotinamide on the microregional heterogeneity of oxygen delivery within a murine tumour. *J. Natl Cancer Inst.*, **82**, 672-676.

CHAPLIN, D.J., TROTTER, M.J., SKOV, K.A. & HORSMAN, M.R. (1990*b*). Modification of tumour radiation response *in vivo* by the benzamide analogue pyrazinamide. *Br. J. Cancer*, **62**, 561-566.

CHAPLIN, D.J., HORSMAN, M.R. & AOKI, D.S. (1991). Nicotinamide, Fluosol-DA and carbogen: a strategy to reoxygenate acutely and chronically hypoxic cells *in vivo*. *Br. J. Cancer*, **63**, 109-113.

CHAPLIN, D.J. & TROTTER, M.J. (1991). Chemical modifiers of tumour blood flow. In *Tumour Blood Supply and Metabolic Microenvironment*. Vaupel, P. & Jain, R.K. (eds). *Gustav Fisher Verlag. Stuttgard.* 65-85.

COURTENAY, V.D. (1976). A soft agar colony assay for Lewis lung tumour and B16 melanoma taken directly from the mouse. *Br. J. Cancer*, **34**, 39-45.

FALK, S.J., WARD, R. & BLEEHAN, N.M. (1992). The influence of carbogen breathing on tumour tissue oxygenation in man evaluated by computerised $pO_2$ histography. *Br. J. Cancer*, **66**, 919-924.

HORSMAN, M.R. (1992). Carbogen and nicotinamide: expectations too high? *Radiotherapy Oncol.*, **24**, 121-122.

HORSMAN, M.R., CHAPLIN, D.J. & OVERGAARD, J. (1990). The combination of nicotinamide and hyperthermia to eliminate radioresistant chronically and acutely hypoxic tumour cells. *Cancer Res.*, **50**, 7430-7436.

JIRTLE, R.E. (1988). Chemical modification of tumour blood flow. *Int. J. Hyperthermia*, **4**, 355-371.

KJELLEN, E., JOINER, M.C., COLLIER, J.M., JOHNS, H. & ROJAS, A.F. (1991). A therapeutic benefit from combining normobaric carbogen or oxygen with nicotinamide in fractionated X-ray treatments. *Radiotherapy. Oncol.*, **22**, 81-91.

MINCHINTON, A.I., DURAND, R.E. & CHAPLIN, D.J. (1990). Intermittent blood flow in the KHT sarcoma flow cytometry studies using Hoechst 33342. *Br. J. Cancer*, **62**, 195-200.

ROCKWELL, S. (1985). The use of perfluorochemical emulsion to improve oxygenation in a solid tumour. *Int. J. Radiat. Oncol. Biol. Phys.*, **11**, 97-103.

ROCKWELL, S., MATE, T.P., IRVIN, C.G. & NIERENBURG, M. (1986). Reactions of tumour and normal tissues in mice to irradiation in the presence and absence of a perfluorochemical emulsion. *Int. J. Radiat. Oncol. Biol. Phys.*, **12**, 1315-1318.

ROJAS, A. (1991). Radiosensitisation with normobaric oxygen and carbogen. *Radiotherapy Oncol. Suppl.*, **20**, 65-70.

ROJAS, A., JOINER, M.C. & DENEKAMP, J. & 4 others (1992). Extrapolations from laboratory and preclinical studies for the use of carbogen and nicotinamide in radiotherapy. *Radiotherapy Oncol.*, **24**, 123-124.

RUBIN, P., HANLEY, J., KEYS, H.M., MARCIAL, V. & BRADY, L. (1979). Carbogen breathing during radiation therapy. *Int. J. Radiat. Oncol. Biol. Phys.*, **5**, 1963-1970.

SASAI, K., ONO, K., NISHIDAI, T. (1989). Variation in tumour response to Fluosol-DA (20%). *Int. J. Radiat. Oncol. Biol. Phys.*, **16**, 1149-1152.

SHELDON, P.W. & HILL, S.A. (1977). Hypoxic cell radiosensitisers and local control by X-ray of a transplanted tumour in mice. *Br. J. Cancer*, **35**, 795-808.

SIEMANN, D.W., HILL, R.P. & BUSH, R.S. (1977). The importance of the pre-irradiation breathing times of oxygen and carbogen (5% $CO_2$ 95%$O_2$) on the *in vivo* radiation response of a murine sarcoma. *Int. J. Radiat. Oncol. Biol. Phys.*, **2**, 903-911.

SONG, C.W., ZHANG, W.L., PENCE, D.M., LEE, I. & LEVITT, S.H. (1985). Increased radiosensitivity of tumours by perfluorochemicals and carbogen. *Int. J. Radiat. Oncol. Biol. Phys.*, **11**, 1833-1836.

SONG, C.W., LEE, I., HASEGAWA, T., RHEE, J.G. & LEVITT, S.H. (1987). Increase in $pO_2$ and radiosensitivity of tumours by Fluosol-DA (20%) and carbogen. *Cancer Res.*, **47**, 442–446.

STRATFORD, M.R.L., ROJAS, A., HALL, D.W., DENNIS, M.F., DISCHE, S., JOINER, M.C. & HODGKISS, R.J. (1992). Pharmacokinetics of nicotinamide and its effect on blood pressure, pulse and body temperature in normal human volunteers. *Radiotherapy & Oncol.*, **25**, 37–42.

TEICHER, B.A. & ROSE, C.M. (1984). Perfluorochemical emulsions can increase tumour radiosensitivity. *Science*, **223**, 934–936.

TROTTER, M.J., CHAPLIN, D.J., DURAND, R.E. & OLIVE, P.L. (1989). The use of fluorescent probes to identify regions of transient perfusion in murine tumours. *Int. J. Radiat. Oncol. Biol. Phys.*, **16**, 931–935.

ZACKHEIM, H.S., VASILY, D.B., WESTPHAL, M.L. & HASTINGS, C.W. (1981). Reactions to nicotinamide. *J. Am. Acad. Dermatol.*, **4**, 736–737.